# Effects of Tea Polyphenols on the Pharmacokinetics of Atorvastatin and Underlying Mechanisms in Hyperlipidemic Rats

**DOI:** 10.1002/fsn3.72543

**Published:** 2026-11-03

**Authors:** Can Feng, Feng Zhao, Dengyu Zuo, Guoxun Pang, Yinling Ma

**Affiliations:** ^1^ School of Pharmacy Hebei Medical University Shijiazhuang China; ^2^ Hebei Key Laboratory of Clinical Pharmacy, Department of Pharmacy Hebei General Hospital Shijiazhuang China

**Keywords:** atorvastatin, food‐drug interactions, hyperlipidemia, pharmacokinetics, tea polyphenols

## Abstract

This study systematically explored the impact of tea polyphenol (TP) on the pharmacokinetics of atorvastatin (ATV) and underlying mechanisms in male Sprague–Dawley rats with hyperlipidemia induced by high‐fat diet. The study was conducted using UHPLC–MS/MS, Western Blot, and in situ single‐pass intestinal perfusion experiments. Pharmacokinetic analysis revealed that high‐dose TP administration (200 mg/kg, 7 days) significantly reduced the *C*_max_ and AUC_0–*t*_ of ATV by 43.83% and 43.38%, respectively, while increasing CL_z_/*F* by 85.04%. Similar reductions were observed for statin metabolites 2‐hydroxy atorvastatin (o‐ATV) and 4‐hydroxy atorvastatin (ρ‐ATV). Western blot results showed that 7‐day TP treatment downregulated CYP3A4 expression but upregulated intestinal P‐gp expression to 1.37‐fold (LDTP) and 1.84‐fold (HDTP). Intestinal perfusion experiments further confirmed that TP decreased the cumulative intestinal absorption amount (*M*) of ATV by 40%. EGCG and ECG exhibited significant inhibitory effects to ATV intestinal absorption, reducing the *M* by 44% and 48%, respectively. In contrast, EGC and EC showed a nonsignificant trend toward slightly increased absorption of ATV. In summary, TP may lower ATV bioavailability through P‐gp‐mediated efflux induction and potential physicochemical interactions, implying caution for long‐term high‐dose intake of TP or supplements.

## Graphical abstract

A study investigated the effects of tea polyphenols (TP) on the pharmacokinetics of atorvastatin (ATV) and the underlying mechanisms in hyperlipidemic rats. Pharmacokinetic analysis and Western blot were performed to measure ATV plasma concentrations and target protein expression, alongside in situ intestinal perfusion assays. The results showed that P‐glycoprotein expression was elevated, and intestinal cumulative absorption of ATV was suppressed. The AUC and *C*_max_ of ATV were decreased, while CL_z_/*F* was increased, thereby reducing its bioavailability.

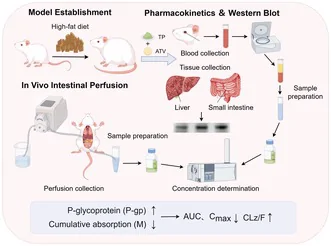

**Abbreviations**
ATV: atorvastatin
CE: collision energy
CLz/*F*
: clearance per unit time
DP: declustering potential
EC: epicatechin
ECG: epicatechin gallate
ECL: enhanced chemiluminescence
EGC: epigallocatechin
EGCG: epigallocatechin gallate
HDL‐C: high‐density lipoprotein cholesterol
HDTP: high‐dose tea polyphenols
HMG‐CoA: 3‐hydroxy‐3‐methylglutaryl coenzyme A
IS: internal standard
LDL‐C: low‐density lipoprotein cholesterol
LDTP: low‐dose tea polyphenols
TC: total cholesterol
TG: triglycerides
TP: tea polyphenols
VER: verapamil hydrochloride
*ο*‐ATV: *ο*‐hydroxy atorvastatin
*ρ*‐ATV: *ρ*‐hydroxy atorvastatin

## 1 Introduction

Hyperlipidemia is a metabolic disorder characterized by elevated levels of total cholesterol, triglycerides, and other lipid components in the blood (Wang et al. 2023). Dyslipidemia, particularly increased low‐density lipoprotein cholesterol (LDL‐C), is a critical factor in atherosclerosis development and a significant risk factor for coronary heart disease (Arvanitis and Lowenstein 2023), cerebral infarction, and damage to other organs (Khoi et al. 2024). In recent years, the prevalence of lipid abnormalities has been rising, increasingly affecting younger individuals and imposing a substantial burden on global health systems.

Atorvastatin (ATV), a cornerstone medication for clinical lipid management, specifically inhibits 3‐hydroxy‐3‐methylglutaryl coenzyme A reductase (HMG‐CoA reductase), effectively blocking the endogenous cholesterol synthesis pathway (Gammal et al. 2025). However, due to its complex pharmacokinetics, drug interactions constitute a critical concern in clinical practice. Following oral administration, ATV is absorbed in the intestine and undergoes extensive hepatic metabolism. As the most abundant CYP isoform in the human liver and intestine, CYP3A4 plays a pivotal role in the oxidative metabolism of ATV, converting it into various metabolites (Adachi et al. 2023; Szkutnik‐Fiedler et al. 2024). Concurrently, P‐glycoprotein (P‐gp), an efflux transporter expressed in the liver, kidneys, and intestines, mediates the excretion of ATV and its metabolites (Wu et al. 2023). P‐gp actively transports substrates into bile and urine, thereby facilitating drug elimination via feces and urine (Matheny et al. 2001; Saaby and Brodin 2017; Skinner et al. 2023). Variations in the activities of these metabolic enzymes or transport proteins can significantly impact ATV disposition, potentially leading to adverse events such as liver dysfunction (Cai et al. 2021), myalgia (Macchi et al. 2022), severe rhabdomyolysis (Montastruc 2023), or reduced therapeutic efficacy, necessitating careful monitoring.

Tea, a globally consumed functional food, has been demonstrated in numerous studies to possess diverse pharmacological properties, including antioxidant (Zhao et al. 2022), hypolipidemic (Ye et al. 2023), and anticancer activities (Trisha et al. 2022). Tea polyphenols (TP) are a class of polyhydroxy phenolic compounds found in all Camellia‐derived tea (green, black and related), with green tea having the highest content (constituting approximately 30% of its dry weight) (Tallei et al. 2021). Its main active components are catechin compounds, including epigallocatechin gallate (EGCG), epigallocatechin (EGC), epicatechin gallate (ECG), and epicatechin (EC) (Kochman et al. 2020). These compounds exhibit potent antioxidant capacity and demonstrate multiple pharmacological activities, including hypolipidemic (Wang et al. 2022), anti‐atherosclerotic (Liu et al. 2024), and hepatoprotective effects (Zhou et al. 2025). Emerging evidence suggests that TP modulates the activity of key metabolic enzymes and transporters, specifically CYP3A, organic anion transporting polypeptide (OATP), and P‐gp (Kondo et al. 2019; Zhang and Qiu 2024). Studies have shown that TP has the potential to interfere with the pharmacokinetics of statins. However, previous research has primarily focused on other statins such as simvastatin and rosuvastatin (Siew‐Keah et al. 2023), leaving the pharmacokinetic profile of atorvastatin still underexplored.

Given the widespread consumption of TP and the established cardiovascular benefits, their concurrent use with ATV is prevalent. However, the impact of the combination on the absorption, metabolism, and excretion of ATV remains unclear in hyperlipidemic organisms. Therefore, this study aims to (i) elucidate the pharmacokinetic effects of combining TP with ATV, and (ii) determine whether TP alters the functions of metabolic enzymes and transporters. The objective is to establish a scientific foundation for the rational consumption of TP.

## 2 Materials and Methods

### 2.1 Chemicals and Reagents

Tea polyphenols, carbamazepine, epigallocatechin gallate, epigallocatechin, epicatechin gallate, and epicatechin were purchased from Shanghai Macklin Biochemical Co. Ltd. (Shanghai, China). Atorvastatin calcium and verapamil hydrochloride were obtained from Aladdin (Shanghai, China). Additionally, ο‐hydroxy atorvastatin and ρ‐hydroxy atorvastatin were purchased from Abmole (Houston, USA). Methanol, acetonitrile, isopropanol, and formic acid were all of LC–MS grade. The antibodies used in this study, including CYP3A4 (18227‐1‐AP), P‐gp (22336‐1‐AP), GAPDH (10494‐1‐AP), and secondary antibody (SA00001‐2), were purchased from Proteintech (Chicago, USA). BCA protein quantification kits were provided by Solarbio Technology Co. Ltd. (Beijing, China). The chromogenic biochemical kits for determining the levels of TC, TG, LDL‐C, and HDL‐C were purchased from Nanjing Jiancheng Bioengineering Institute (Nanjing, Jiangsu, China).

### 2.2 Animals

SPF‐grade male Sprague–Dawley rats (weight 150–170 g) were obtained from the Experimental Animal Center of Hebei Medical University (China). The animals were housed under controlled conditions: temperature 23°C ± 2°C; relative humidity 55% ± 5%, and a 12‐h light/dark cycle. All experimental procedures and animal welfare protocols adhered to the guidelines of the Committee for the Management of Experimental Animals at Hebei Provincial People's Hospital (Approval No. 2024‐DW‐068). Hyperlipidemia was induced by feeding the rats a high‐fat diet composed of 12% lard, 10% sucrose, 10% egg yolk, 1% cholesterol, 0.5% sodium cholate, and 66.5% basal chow for 3 weeks. Serum lipid levels(TC, TG, LDL‐C, and HDL‐C) were measured using commercial assay kits to monitor the progression of the model.

### 2.3 LC–MS/MS Quantitative Analysis Method

A UHPLC–MS/MS method was developed to simultaneously quantify ATV and its metabolites, ρ‐ATV and ο‐ATV, in rat plasma, employing carbamazepine as the internal standard (IS). Chromatographic separation was performed on a Shimadzu LC‐20A system equipped with an Agilent C_18_ column (2.7 μm, 2.1 mm × 100 mm). The mobile phase consisted of water containing 0.1% formic acid (A) and acetonitrile containing 0.1% formic acid (B), delivered at a flow rate of 0.4 mL/min. The gradient elution program was as follows: 0–2 min, 45% B; 2–2.1 min, 45%–55% B; 2.1–6 min, 55% B. The total run time was 6.0 min, which included a 1 min pre‐column equilibration. The column temperature was maintained at 40°C, and the injection volume was 5 μL. The retention times of IS, ρ‐ATV, ο‐ATV, and ATV were 1.34, 1.63, 3.50, and 3.86 min, respectively.

Mass spectrometric analysis was conducted using an AB SCIEX 5500 instrument equipped with an electrospray ionization (ESI) source. The optimized mass‐to‐charge (m/z) transitions were monitored as follows: 559.5 → 440.5 for ATV, 237.1 → 194.1 for carbamazepine (IS), and 575.2 → 440.3 for both ο‐ATV and ρ‐ATV. The declustering potential (DP) was set to 130 V for ATV, 120 V for the IS, and 91 V for both metabolites. The collision energy (CE) was optimized to 30 eV for ATV, 13 eV for the IS, and 33 eV for both ο‐ATV and ρ‐ATV.

### 2.4 Effect of TP on the Pharmacokinetics of ATV

#### 2.4.1 Animal Grouping and Dosing Regimen

A total of 30 hyperlipidemic rats were fasted for 12 h prior to the experiment but allowed free access to water. The rats were randomly assigned to five groups (*n* = 6 per group) and administered treatments via oral gavage as follows: Group I received 0.5% carboxymethyl cellulose sodium (CMC‐Na) solution for seven consecutive days, followed by a single dose of ATV on the final day; Group II received 0.5% CMC‐Na for six consecutive days, followed by a single coadministration of TP (100 mg/kg) and ATV on the last day; Group III received 0.5% CMC‐Na for six consecutive days, followed by a single coadministration of TP (200 mg/kg) and ATV on the last day; Group IV received TP (100 mg/kg) for seven consecutive days, followed by a single dose of ATV on the seventh day; and Group V received TP (200 mg/kg) for seven consecutive days, followed by a single dose of ATV on the seventh day. ATV was also formulated as a suspension in 0.5% CMC‐Na. A single dose of ATV (1.8 mg/kg) was administered 5 min after the gavage of either 0.5% CMC‐Na or TP solution in all groups.

#### 2.4.2 Sample Collection and Processing

Blood samples (approximately 0.2 mL) were collected from the ophthalmic venous plexus at the following time points post‐ATV administration: 0, 0.083, 0.166, 0.33, 0.5, 0.75, 1, 1.5, 2, 4, 6, 8, 12, and 24 h. Samples were immediately transferred to heparinized tubes and centrifuged at 11,500 × *g* and 4°C for 5 min. For protein precipitation, 50 μL of plasma was mixed with 5 μL of aqueous formic acid, 10 μL of carbamazepine (internal standard), and 140 μL of methanol. The mixture was vortexed for 2 min and centrifuged under the same conditions (11,500 × *g*, 4°C) for 5 min. Finally, 30 μL of the supernatant was transferred into an autosampler vial for the quantitative analysis of ATV and its metabolites.

#### 2.4.3 Data Analysis

Pharmacokinetic parameters, including maximum plasma concentration (*C*_max_), time to peak (*T*_max_), area under the drug‐time curve (AUC_0–*t*_), elimination half‐life (*t*_1/2_), and oral clearance (CL_z_/*F*), were calculated using DAS software (version 3.2.8). Statistical analyses were performed using SPSS software, and intergroup differences were assessed by one‐way ANOVA.

### 2.5 Western Blot Analysis

After the experiment, rat liver and intestine were quickly collected, washed with phosphate‐buffered saline (PBS), blotted dry with filter paper, and stored at −80°C. Tissue samples were weighed and homogenized in RIPA lysis buffer containing protease inhibitor (100:1 ratio). The homogenate was centrifuged (11,500 × *g*, 4°C) for 20 min, and the amount of protein in the supernatant was determined by BCA assay. Samples were mixed with 5× loading buffer, denatured at 90°C for 10 min, and stored at −80°C until further use. Equal amounts of proteins were separated by 12% SDS‐PAGE and subsequently transferred to a PVDF membrane via wet transfer. The membranes were blocked with 5% skim milk in TBST for 2 h at room temperature, followed by three TBST washes. Primary antibodies were diluted in 5% BSA solution at the following ratios: CYP3A4 antibody (1:2500), P‐gp antibody (1:1000), and GAPDH antibody (1:17,500). Target protein bands were excised according to molecular weight and incubated with primary antibodies overnight at 4°C. The secondary antibody (diluted 1:6000) was applied for 2 h at room temperature. Protein bands were visualized using enhanced chemiluminescence (ECL) and quantified using ImageJ software.

### 2.6 In Situ Intestinal Absorption Study

#### 2.6.1 Preparation of Drug Solutions

Krebs‐Ringer's (K‐R) buffer was prepared by dissolving 7.8 g NaCl, 0.35 g KCl, 0.37 g CaCl_2_, 0.02 g MgCl_2_, 0.32 g NaH_2_PO_4_, 1.37 g NaHCO_3_, and 1.4 g D‐glucose in 1000 mL of water. For the blank perfusion, K‐R solution without drugs was pumped into the intestinal segment. The drug‐containing perfusion fluid was prepared by diluting an appropriate amount of ATV stock solution with K‐R buffer to achieve a final concentration of 30 μg/mL. Mixed perfusion fluids were further prepared by adding verapamil hydrochloride (VER, 0.1 mmol/L), TP (100 μg/mL), or individual catechins (EGCG, EGC, ECG, and EC, 50 μmol/L) to the 30 μg/mL atorvastatin solution.

#### 2.6.2 Single‐Pass Intestinal Perfusion

SPF‐grade male rats (approximately 6–8 weeks old) were fed a high‐fat diet to establish the model. A total of 28 model rats were randomly assigned to seven groups: ATV, VER + ATV, TP + ATV, EGCG + ATV, EGC + ATV, ECG + ATV, and EC + ATV. Rats were anesthetized with 20% urethane (5 mL/kg). After a midline laparotomy (3–4 cm), approximately 10 cm of intestine was isolated 20 cm distal to the pylorus. The intestinal contents were flushed with 37°C physiological saline. The segment was perfused with 37°C test solution at a flow rate of 0.2 mL/min. The effluent was collected at the outflow site. Following a 20‐min equilibration period, perfusate was collected in tubes changed every 15 min for a total duration of 120 min. At the conclusion of the experiment, the length and radius of the intestinal segment were measured.

#### 2.6.3 Data Computation and Analysis

The absorption rate constant (*K*_a_, /min), effective permeability coefficient (*P*_eff_, cm/min), and cumulative absorption amount (*M*, μg/cm^2^) were calculated using the following equations (Wu et al. 2024):

$$K_{a}=Q\cdot \left(1-C_{\text{out}}/C_{\text{in}}\cdot V_{\text{out}}/V_{\text{in}}\right)/{\mathrm{\pi r}}^{2}L$$

$$P_{\mathrm{eff}}=-Q\cdot \ln\;\left(C_{\text{out}}/C_{\text{in}}\cdot V_{\text{out}}/V_{\text{in}}\right)/2\mathrm{\pi rL}$$

$$M=\mathrm{Qt}\cdot \left(C_{\text{in}}-C_{\text{out}}\cdot V_{\text{out}}/V_{\text{in}}\right)/2\mathrm{\pi rL}$$

where *Q* is the perfusion rate (0.20 mL/min); *C*_in_ and *C*_out_ represent the atorvastatin concentrations at the inlet and outlet, respectively (μg/mL); *V*_in_ and *V*_out_ are the volumes of perfusate entering and exiting the system, respectively (mL); *r* is the radius, and *L* is the length of the experimental intestinal segment (cm).

## 3 Results

### 3.1 Pharmacokinetic Interaction Between ATV and TP

The pharmacokinetic alterations of ATV and its major metabolites (ο‐ATV and ρ‐ATV) following single and multiple co‐administration with TP were analyzed by UHPLC‐MS/MS (0–24 h), as illustrated in Figure 1. All pharmacokinetic parameters are summarized in Tables 1, 2, 3. Initially, this study assessed the effects of single co‐administration of TP at doses of 100 and 200 mg/kg on ATV's pharmacokinetics. Both TP doses reduced systemic exposure of ATV, with more pronounced effects at the higher dose (200 mg/kg, *p* < 0.05), resulting in significant reductions of 28.91% and 32.34% in AUC_0–*t*_ and *C*_max_, respectively, whereas the other pharmacokinetic parameters showed no significant changes. Consistently, similar decreases in peak concentrations and plasma exposure were observed for metabolites ο‐ATV and ρ‐ATV. To simulate chronic tea consumption, a 7‐day TP pre‐treatment (200 mg/kg) followed by ATV administration was further investigated. Prolonged TP exposure markedly enhanced the interaction, resulting in greater reductions in ATV exposure (43.83% decrease in *C*_max_, 43.38% decrease in AUC_0–t_ *)* along with an 85.04% increase in clearance. Metabolite ο‐ATV showed decreased exposure (37.60% lower *C*_max_ and 29.52% lower AUC_0–*t*_) with a 30.43% elevation in clearance. Similarly, ρ‐ATV exhibited a 52.57% reduction in *C*_max_ and a 27.90% decrease in AUC_0–*t*_, with prolonged *T*_max_ (increased from 0.71 to 1.42 h) and increased clearance. These findings indicate that 7‐day pre‐treatment with TP significantly affected CL_z_/F and AUC_0–*t*_ at both doses, while significant reduction in *C*_max_ was only observed for the high‑dose TP group, but not the other measures.

**FIGURE 1:**
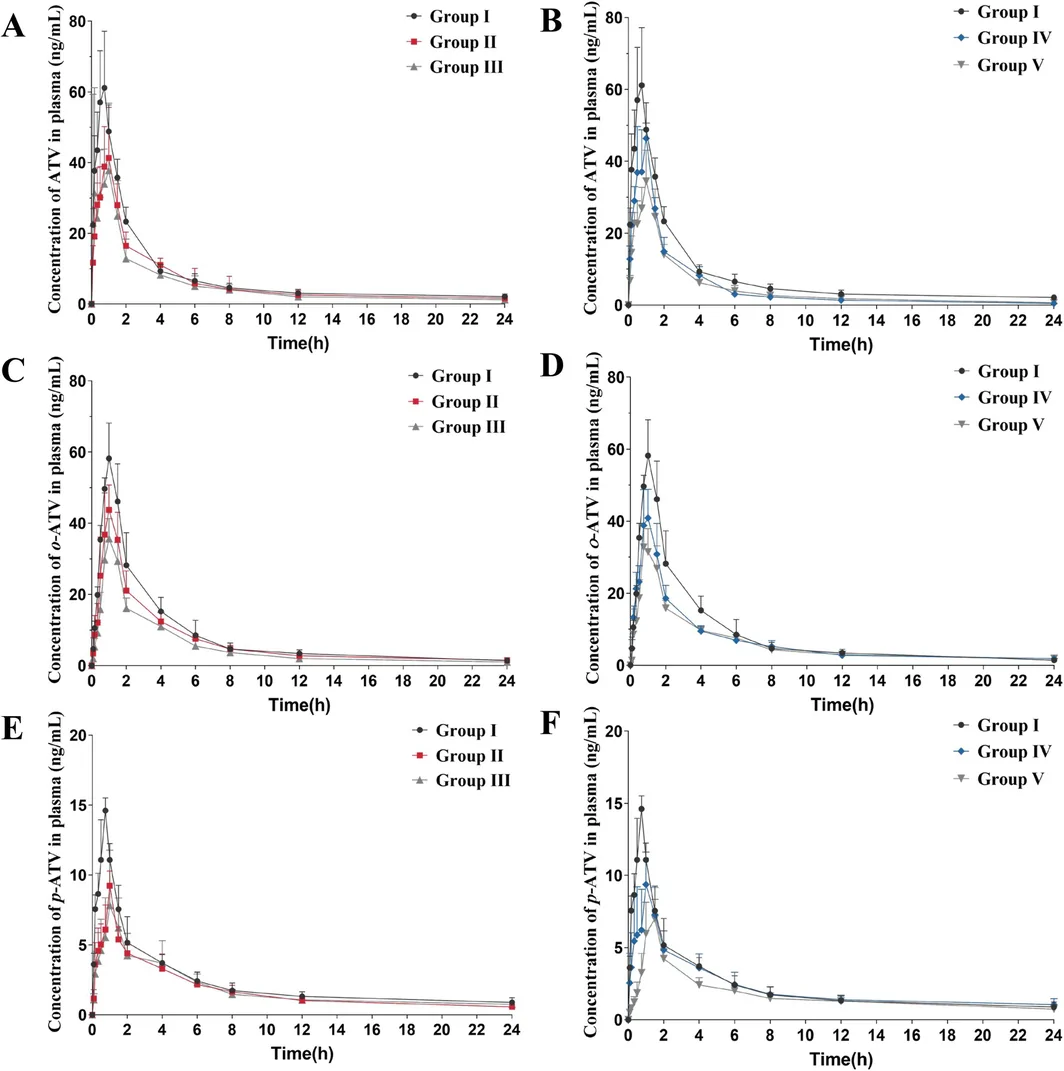
Plasma concentration‐time curves of ATV (A, B), ο‐ATV (C, D), and ρ‐ATV (E, F) in hyperlipidemic rats. Group I represents rats given 7 days of a 0.5% CMC‐Na and 1.8 mg/kg ATV on the last day. Group II rats were given 6 days of a 0.5% CMC‐Na; both 100 mg/kg TP and 1.8 mg/kg ATV were given on the last day. Group III rats were given 6 days of a 0.5% CMC‐Na; both 200 mg/kg TP and 1.8 mg/kg ATV were given on the last day. Group IV rats were given 100 mg/kg TP for seven consecutive days and 1.8 mg/kg of ATV on the 7th day. Group V rats were given 200 mg/kg TP for seven consecutive days and 1.8 mg/kg of ATV on the 7th day. *n* = 6 of each group. Data are presented as mean ± SD.

**TABLE 1:** Pharmacokinetic parameters of ATV in hyperlipidemic rats.

Parameters (unit)	Group I	TP single‐day administration		TP 7‐day administration	
		Group II	Group III	Group IV	Group V
AUC_0–*t*_ (ng/mL*h)	189.03 ± 34.75	159.86 ± 37.30	134.38 ± 24.87*	118.26 ± 19.34**	107.02 ± 21.40***
*t*_1/2_ (h)	6.65 ± 3.62	7.73 ± 3.81	7.74 ± 2.42	6.34 ± 1.17	7.62 ± 2.61
*T*_max_ (h)	0.75 ± 0.16	0.85 ± 0.27	0.96 ± 0.10	0.88 ± 0.21	0.83 ± 0.20
CL_z_/*F* (L/h/kg)	97.62 ± 20.25	120.64 ± 34.88	127.35 ± 34.90	167.43 ± 27.12**	180.64 ± 39.34**
*C*_max_ (ng/mL)	64.91 ± 15.86	51.45 ± 10.87	43.92 ± 14.29*	48.78 ± 7.39	36.46 ± 7.86**

*Note:* Group I represents rats given 7 days of a 0.5% CMC‐Na and 1.8 mg/kg ATV on the last day. Group II rats were given 6 days of a 0.5% CMC‐Na; both 100 mg/kg TP and 1.8 mg/kg ATV were given on the last day. Group III rats were given 6 days of a 0.5% CMC‐Na; both 200 mg/kg TP and 1.8 mg/kg ATV were given on the last day. Group IV rats were given 100 mg/kg TP for seven consecutive days and 1.8 mg/kg of ATV on the 7th day. Group V rats were given 200 mg/kg TP for seven consecutive days and 1.8 mg/kg of ATV on the 7th day. *p < 0.05, **p < 0.01, ***p < 0.001, versus Group I.

**TABLE 2:** Pharmacokinetic parameters of ο‐ATV in hyperlipidemic rats.

Parameters (unit)	Group I	TP single‐day administration		TP 7‐day administration	
		Group II	Group III	Group IV	Group V
AUC_0–*t*_ (ng/mL*h)	202.04 ± 41.85	163.24 ± 22.08	127.35 ± 21.96**	155.91 ± 18.56*	142.40 ± 15.18**
*t*_1/2_ (h)	10.13 ± 2.62	8.89 ± 6.52	7.20 ± 3.54	5.92 ± 2.36	13.24 ± 14.48
*T*_max_ (h)	0.92 ± 0.13	0.96 ± 0.10	0.96 ± 0.10	0.92 ± 0.13	0.88 ± 0.14
CL_z_/*F* (L/h/kg)	91.97 ± 14.92	111.22 ± 5.33	149.59 ± 25.64**	123.51 ± 19.84	119.96 ± 32.03
*C*_max_ (ng/mL)	58.68 ± 9.25	46.89 ± 6.95	38.49 ± 3.28***	47.58 ± 10.93*	36.62 ± 4.21***

*Note:* Group I represents rats given 7 days of a 0.5% CMC‐Na and 1.8 mg/kg ATV on the last day. Group II rats were given 6 days of a 0.5% CMC‐Na; both 100 mg/kg TP and 1.8 mg/kg ATV were given on the last day. Group III rats were given 6 days of a 0.5% CMC‐Na; both 200 mg/kg TP and 1.8 mg/kg ATV were given on the last day. Group IV rats were given 100 mg/kg TP for seven consecutive days and 1.8 mg/kg of ATV on the 7th day. Group V rats were given 200 mg/kg TP for seven consecutive days and 1.8 mg/kg of ATV on the 7th day. *p < 0.05, **p < 0.01, ***p < 0.001, versus Group I.

**TABLE 3:** Pharmacokinetic parameters of ρ‐ATV in hyperlipidemic rats.

Parameters (unit)	Group I	TP single‐day administration		TP 7‐day administration	
		Group II	Group III	Group IV	Group V
AUC_0–*t*_ (ng/mL*h)	56.37 ± 7.22	43.03 ± 6.29	44.33 ± 12.34	52.45 ± 8.96	40.64 ± 3.45*
*t*_1/2_ (h)	12.52 ± 4.06	9.32 ± 3.74	10.62 ± 4.19	19.54 ± 24.01	14.51 ± 11.36
*T*_max_ (h)	0.71 ± 0.10	0.96 ± 0.10	1.17 ± 0.26**	0.92 ± 0.20	1.42 ± 0.20***
CL_z_/*F* (L/h/kg)	277.16 ± 56.10	406.32 ± 60.07	391.37 ± 101.26	287.69 ± 94.09	380.88 ± 107.86
*C*_max_ (ng/mL)	14.99 ± 2.15	9.23 ± 1.05**	8.49 ± 3.18**	9.78 ± 2.55*	7.11 ± 1.55***

*Note:* Group I represents rats given 7 days of a 0.5% CMC‐Na and 1.8 mg/kg ATV on the last day. Group II rats were given 6 days of a 0.5% CMC‐Na; both 100 mg/kg TP and 1.8 mg/kg ATV were given on the last day. Group III rats were given 6 days of a 0.5% CMC‐Na; both 200 mg/kg TP and 1.8 mg/kg ATV were given on the last day. Group IV rats were given 100 mg/kg TP for seven consecutive days and 1.8 mg/kg of ATV on the 7th day. Group V rats were given 200 mg/kg TP for seven consecutive days and 1.8 mg/kg of ATV on the 7th day. *p < 0.05, **p < 0.01, ***p < 0.001, versus Group I.

### 3.2 Effects of TP on CYP3A4 and P‐gp Expression Levels

Western blot analysis was performed to investigate TP's modulatory effects on CYP3A4 and P‐gp in Figure 2. Long‐term TP treatment (100 and 200 mg/kg) significantly inhibited the expression of CYP3A4 in the liver and intestine. In the liver, HDTP reduced CYP3A4 expression levels to 0.33‐fold of the control group, while LDTP reduced it to 0.67‐fold. In the intestine, LDTP and HDTP decreased CYP3A4 expression to 0.74‐ and 0.42‐fold, respectively. Conversely, the study results indicate that TP consistently upregulated the expression of efflux transporter P‐gp. In the intestine, the expression level of P‐gp in the LDTP group was approximately 1.37‐fold, and in the HDTP group, it was upregulated to 1.84‐fold compared to the control. Similarly, hepatic P‐gp expression was elevated to 1.21‐fold (LDTP) and 1.34‐fold (HDTP).

**FIGURE 2:**
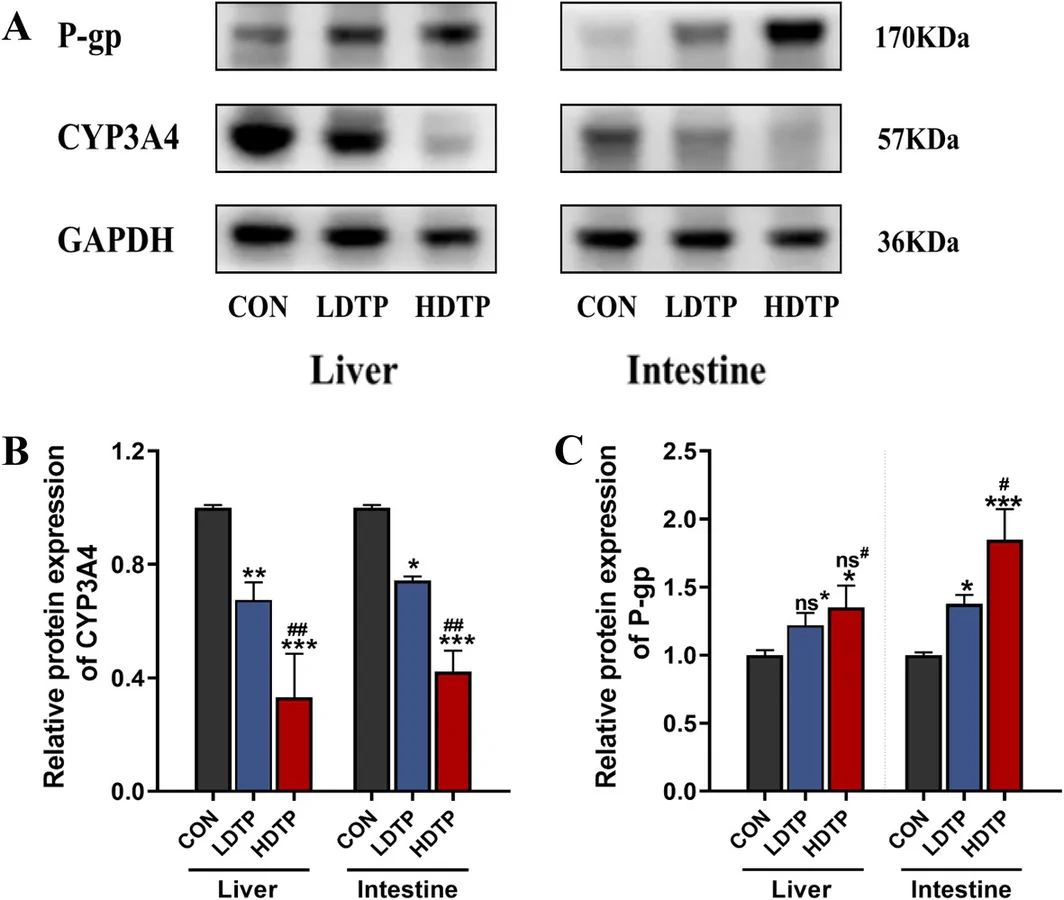
Effects of TP on the expression of CYP3A4 and P‐gp in the liver and intestine. (A) Representative protein blot images of control and tea polyphenol‐treated groups. (B) CYP3A4 protein quantitative expression. (C) P‐gp protein quantitative expression. Rats in the CON group represent the 1.8 mg/kg ATV administration group. Rats in the LDTP group are the 100 mg/kg TP and 1.8 mg/kg ATV coadministration groups. Rats in the HDTP group were coadministered with 200 mg/kg TP and 1.8 mg/kg ATV. *n* = 3 of each group. Data are presented as mean ± SD. Data were compared with the CON group by one‐way ANOVA, *p < 0.05, **p < 0.01, ***p < 0.001; one‐way ANOVA statistics versus the LDTP group, ^#^ *p* < 0.05, ^##^ *p* < 0.01, ^###^ *p* < 0.001.

### 3.3 Effects of TP and Catechins on the Intestinal Absorption of Atorvastatin

Using an in situ single‐pass intestinal perfusion model in rats, the effects of TP, VER, and different catechins on ATV absorption were investigated. The absorption rate constant (*K*_a_), effective permeability coefficient (*P*_eff_), and cumulative absorption amount (*M*) are detailed in Table 4 and Figure 3. Coadministration with the classical P‐gp inhibitor VER significantly enhanced ATV absorption (*M* = 25.56 μg/cm^2^), confirming ATV as a P‐gp substrate. Conversely, compared to the ATV group (*K*_a_ = 1.11 × 10^−2^/min, *P*_eff_ = 1.05 × 10^−3^ cm/min, *M* = 19.93 μg/cm^2^), the TP + ATV group inhibited ATV absorption. This was accompanied by nonsignificant decreases in *K*_a_ and *P*_eff_ (*p* > 0.05), with the intestinal accumulation markedly dropping to 12.01 μg/cm^2^ (*p* < 0.05). The effects of catechins varied significantly: in the EGCG + ATV group, all three parameters were significantly reduced: *K*_a_ by 56% (*p* < 0.05), *P*_eff_ by 50% (*p* < 0.05), and *M* to 11.11 μg/cm^2^ (−44%, *p* < 0.01). The ECG + ATV group also exhibited an inhibitory effect on absorption, with *M* decreasing to 10.33 μg/cm^2^ (*p* < 0.01), representing a reduction of approximately 48%. However, there were no significant changes in *K*_a_ and *P*_eff_ (*p* > 0.05). In contrast, the EGC + ATV and EC + ATV groups had minimal impacts on ATV absorption parameters (*K*_a_, *P*_eff_, and *M*), although both groups showed nonsignificant trends toward slightly higher absorption (*p* > 0.05).

**TABLE 4:** Absorption parameters of ATV in intestinal perfusion experiments.

Group	Absorption parameters		
	*K*_a_ /×10^−2^ (/min)	*P*_eff_ /×10^−3^ (cm/min)	*M* (μg/cm^2^)
ATV	1.11 ± 0.09	1.05 ± 0.11	19.93 ± 1.54
VER + ATV	1.42 ± 0.15	1.42 ± 0.28	25.56 ± 2.63*
TP + ATV	0.67 ± 0.27	0.60 ± 0.33	12.01 ± 4.91*
EGCG + ATV	0.49 ± 0.18*	0.53 ± 0.14*	11.11 ± 2.47**
EGC + ATV	1.30 ± 0.08	1.25 ± 0.14	23.39 ± 1.41
ECG + ATV	0.69 ± 0.47	0.76 ± 0.30	10.33 ± 4.16**
EC + ATV	1.23 ± 0.09	0.96 ± 0.10	22.08 ± 1.57

*Note:* The Group ATV received a perfusion solution containing only ATV. The Group VER + ATV was administered a perfusion solution containing a mixture of verapamil and ATV. Similarly, the Group TP + ATV received a perfusion solution with a mixture of TP and ATV. For the individual flavonoids, the Groups EGCG/EGC/ECG/EC + ATV represent the use of a perfusion solution containing each respective flavonoid combined with ATV. *p < 0.05, **p < 0.01, versus Group ATV.

**FIGURE 3:**
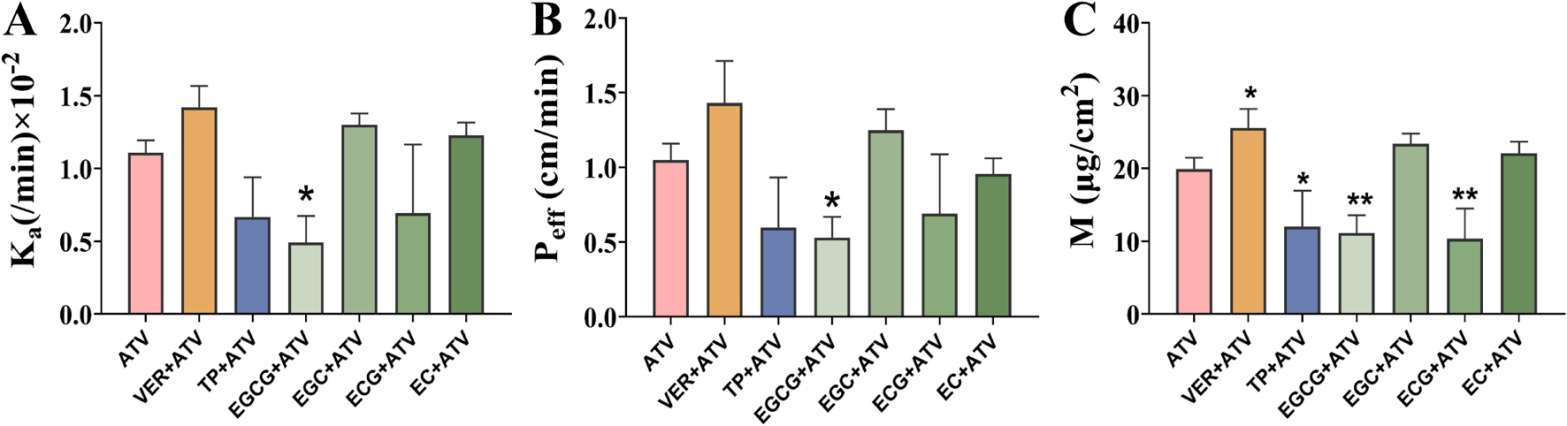
The absorption parameters of ATV in each group of intestinal perfusion. (A) Absorption rate constant (*K*_a_, /min). (B) Effective permeability coefficient (*P*_eff_, cm/min). (C) Cumulative absorption amount (*M*, μg/cm^2^). *n* = 4, data are presented as mean ± SD. Data were compared with the ATV group by one‐way ANOVA, *p < 0.05, **p < 0.01. The Group ATV received a perfusion solution containing only ATV. The Group VER + ATV was administered a perfusion solution containing a mixture of verapamil and ATV. Similarly, the Group TP + ATV received a perfusion solution with a mixture of TP and ATV. For the individual flavonoids, the Groups EGCG/EGC/ECG/EC + ATV represent the use of a perfusion solution containing each respective flavonoid combined with ATV.

## 4 Discussion

A chronic high‐fat diet induces pathological liver injury through lipotoxicity triggered by lipid accumulation (Li et al. 2023; Liu et al. 2023). Tea, commonly employed as a dietary adjunct for lipid‐lowering therapy, demonstrates potential benefits encompassing oxidative stress reduction (Song et al. 2019), protection against free radical‐induced hepatocyte damage (Chen et al. 2022), hepatitis inhibition (Ding et al. 2023; Yang et al. 2024), and lipid‐lowering effects via potent inhibition of HMG‐CoA reductase and SREBP1c (Kanu et al. 2024), alongside promotion of lipid clearance (Zhou et al. 2014).

The concentration of tea polyphenols varies significantly based on tea origin and the extraction process. This study utilized commercially sourced TP extracts, with dosage formulated based on prior scientific hypotheses (Wang et al. 2020): A 70 kg adult consuming two cups of green tea (approximately containing 6–8 g of tea leaves) would ingest ≈2.1 g TP, which translates to a 200 mg/kg dose for rats—a level that is indeed higher than the typical daily tea consumption of the general population. However, published studies have employed similar or even higher doses of TP (Misaka et al. 2013; Maher et al. 2017). Furthermore, considering the low oral bioavailability of TP, a relatively high dose is required to achieve sufficient systemic concentrations necessary to observe their effects on enzymes or transporters, which is a common design strategy in pharmacokinetic interaction studies. Furthermore, the 200 mg/kg dose can be appropriately employed to simulate scenarios involving heavy tea consumers or the ingestion of tea polyphenol dietary supplements. A lower dose of 100 mg/kg was included, which to some extent accounts for the natural variations in tea polyphenol intake under real‐world conditions, including polyphenol dilution during steeping and individual tea consumption habits.

Pharmacokinetic analyses revealed TP influences ATV's in vivo disposition, but the precise mechanism remained obscure from pharmacokinetic parameter shifts alone. Consequently, further exploration was necessitated to elucidate how this complex polyphenol mixture regulates key metabolic enzymes and transporters. WB demonstrated long‐term TP administration suppressed metabolic enzyme CYP3A4 expression, theoretically retarding ATV metabolism and elevating plasma concentrations. However, pharmacokinetic data exhibited synchronous decreases in ATV and its metabolite's AUC and *C*_max_, implying another dominant regulatory mechanism overriding metabolic inhibition. This observation aligns with substantial P‐gp upregulation by TP (1.84‐fold in intestine; 1.34‐fold in liver). Elevated levels of P‐gp expression can mediate the efflux of ATV from intestinal cells into the intestinal lumen, reducing ATV intestinal absorption and bioavailability. Based on our current findings and previous literature indicating that TP may affect the pharmacokinetics of ATV, we hypothesize that the intestine is the primary site of this interaction.

Oral drugs are initially absorbed in the small intestine and then enter systemic circulation (Pathomthongtaweechai and Muanprasat 2021). In situ single‐pass intestinal perfusion is an experimental method that simulates the intraluminal environment of the intestine, precisely focusing on intestinal function while excluding interference from other organs (López‐Yerena et al. 2021). It is commonly used to evaluate the intestinal absorption kinetics of drugs. Our experiments found that TP significantly inhibited ATV cumulative absorption, as indicated by a reduction in *K*_a_ and *P*_eff_, which suggests a slower absorption rate and diminished transmembrane capacity. TP is a mixture containing anthocyanins, phenolic acids, flavonoids, and dominant catechins (≈50%) (EFSA Panel on Food Additives and Nutrient Sources added to Food [ANS] et al. 2018; Trisha et al. 2022), with EGCG comprising 50%–75% of catechins, followed by EGC, ECG, and EC (Capasso et al. 2025). At equivalent concentrations, EGCG and ECG markedly decreased the *M* of atorvastatin, while EGC and EC mildly enhanced absorption. Roth et al. (2011) similarly reported EGCG and ECG's concentration‐dependent inhibition of OATP1A2/OATP2B1‐mediated drug uptake. In vitro studies indicate gallated catechins (EGCG, ECG) more potently modulate drug transport, metabolism, and solubility compared to non‐gallated counterparts (EC, EGC) (Roth et al. 2011; Ge et al. 2011; Ikeda et al. 2012; Kobayashi et al. 2014; Ogawa et al. 2016), showcasing superior OATP inhibitory capacity.

Additionally, literature reports suggest TP directly binds to drugs, altering structure, solubility, and dissociation (Oyanna et al. 2024; Ikeda et al. 2022). As polyhydroxyphenolic compounds, catechins may form insoluble complexes with ATV in the gastrointestinal tract through hydrogen bonding and hydrophobic interactions. This suggests that the effect of tea polyphenols on atorvastatin absorption is not the result of a single factor but may be regulated by multiple mechanisms, including passive diffusion, active transport, and physicochemical interactions.

While this study used a rat model, human trials also confirm TP‐mediated food‐drug interactions. Abdelkawy et al. (2020) found that 300 and 600 mg green tea extract doses reduced ATV's *C*_max_ and AUC_0–∞_ by ~20% while increasing CL_z_/*F* by ~30% in healthy volunteers. Currently, no studies report whether these interactions show complex dynamic changes under pathological conditions. Thus, patients taking long‐term, high‐dose TP or supplements, especially those rich in EGCG and ECG, should exercise caution.

## Author Contributions

**Can Feng:** conceptualization, methodology, investigation, data curation, writing – original draft, writing – review and editing. **Feng Zhao:** conceptualization, validation, funding acquisition, project administration, supervision. **Dengyu Zuo:** data curation, methodology, investigation. **Guoxun Pang:** conceptualization, validation, funding acquisition, project administration. **Yinling Ma:** conceptualization, data curation, funding acquisition, project administration, supervision, visualization, validation, writing – review and editing.

## Funding

This work was financially supported by the Hebei Provincial Key Medical Research Project (No. 20241004), the Hebei Provincial Government Funded Clinical Medicine Excellence Talent Project (No. ZF2023178), the National Natural Science Foundation of China (No. 82104286), and the Natural Science Foundation of Hebei Province (No. H2026307020).

## Conflicts of Interest

The authors declare no conflicts of interest.

## Data Availability

The data that support the findings of this study are available from the corresponding author upon reasonable request.
